# *dld-1* suppression attenuates the detrimental effects of amyloid beta deposition in a *Caenorhabditis elegans* model of Alzheimer’s disease

**DOI:** 10.1186/1750-1326-8-S1-P3

**Published:** 2013-09-13

**Authors:** Waqar Ahmad, Paul Ebert

**Affiliations:** 1 School of Biological Sciences, St Lucia, Brisbane, Australia

## Background

Amyloid beta (Aβ) aggregation is well studied as a major marker and determinant of Alzheimer’s disease (AD) pathology. While less work has been carried out on the role of energy metabolism in AD, there is good evidence that it also contributes to the disease. For example, low metabolic rate and ATP levels are also correlated with AD, which also extends to specific enzymes, metabolites, and proteins associated with glycolysis and the TCA cycle. Dihydrolipoamide dehydrogenase (DLD-1), the subject of this study, is a core metabolic enzyme with specific sequence variants that are associated with increased risk of late onset AD. DLD-1 contributes to four major metabolic multi-enzyme complexes, including *a*-ketoglutarate dehydrogenase (KGDH). A non-DLD subunit of KGDH also has variants that are associated with AD. Additionally, the activity level of the enzyme is significantly inversely correlated with the disease state in humans.

## Materials and methods

We used a previously published *C*. *elegans* model of AD that consists of two strains CL2006 and CL4176, which produce human amyloid beta (1-42) in body-wall muscle cells either constitutively or by temperature upshift, respectively. Aβ aggregation results in muscle impairment, observed as paralysis, which is particularly apparent when a muscle stimulant is applied. Using this assay, we chemically or genetically modified the function or abundance of DLD-1, to determine whether this could modify disease progression or remission. We also modified other metabolic functions to see whether the observed effect was specific to DLD.

## Results

Expression of human Aβ caused significant paralysis of *C. elegans* in the presence of each of the stimulants, aldicarb and serotonin (5-HT). This effect was completely reversed when the dld-1 gene was suppressed in either the constitutive or the inducible Aβ producing strain. The same effect was observed with chemicals that inhibited metabolic pathways involving DLD-1, but not with an uncoupler of the mitochondrial electron transport chain that depletes ATP production capacity. Thus, it was not energy depletion, per se, that caused the effect. Rather, the critical factor seems to be inhibition of an, as yet loosely defined, DLD-1 containing metabolic pathway. The chemicals that were effective in alleviating the Aβ mediated pathology in the model also decreased Aβ aggregation. Interestingly, one of the chemicals in particular effectively decreased Aβ aggregation and reduced the behavioural pathology regardless of whether the Aβ was pre-expressed or co-expressed with the chemical treatment.

## Conclusions

Our results show that inhibition of DLD-1 in our model has protective effects against Aβ toxicity although the precise mechanism is not yet fully understood.

